# Fundus Stretch Index: A Centile-Based Retinal Measure of Myopia

**DOI:** 10.1016/j.xops.2026.101162

**Published:** 2026-03-18

**Authors:** Fabian Yii

**Affiliations:** Robert O Curle Ophthalmology Suite, Institute for Regeneration and Repair, The University of Edinburgh, Edinburgh, UK; Centre for Clinical Brain Sciences, Institute for Neuroscience and Cardiovascular Research, The University of Edinburgh, Edinburgh, UK; Centre for Population Health Sciences, Usher Institute, The University of Edinburgh, Edinburgh, UK; Department of Twin Research and Genetic Epidemiology, King’s College London, London, UK

**Keywords:** Myopia, Fundus stretch index, Fundus refraction offset, Retinal detachment, Glaucoma

## Abstract

**Objective:**

To propose and evaluate the risk-stratification potential of Fundus Stretch Index (FunSI), a centile-based retinal metric that quantifies the geometric deviation of the fundus from what is expected for a given spherical equivalent refraction (SER).

**Design:**

A population-based cohort study.

**Participants:**

Phakic eyes of 25 222 adults (SER: 0 D to –12 D) with adequate-quality fundus photographs at baseline (2009–2010), individually linked to routinely collected health records until 2022.

**Methods:**

Quantile regression was used to estimate the 5th to 95th centiles of 10 dimensionless fundus imaging features (e.g., arterial/venous tortuosity and disc tilt), conditioning on SER. Fundus Stretch Index was computed as the sum of an eye’s SER-specific centile position across all imaging features, normalized to 0-1, such that an eye consistently ranked in the worst centile (fifth or 95th, depending on the direction of change as myopia increased) for its SER would have a value of 1. Cox regression was used to test the association between baseline FunSI and the risk of rhegmatogenous retinal detachment (RD), adjusting for baseline SER, age, sex, ethnicity, Townsend deprivation index, diabetes, hypertension, and ocular trauma; and primary open-angle glaucoma (POAG), adjusting for the same baseline covariates (excluding ocular trauma) plus intraocular pressure and corneal hysteresis.

**Main Outcome Measures:**

Rhegmatogenous RD and POAG onset.

**Results:**

A total of 25 030 and 24 835 adults aged 40-69 years without any prior history of RD/breaks and glaucoma of any subtype were analyzed, respectively. The event rates (new cases per 10 000 person-years) were 4.9 for rhegmatogenous RD and 15.0 for POAG. Higher baseline FunSI was associated with an increased risk of developing rhegmatogenous RD (adjusted hazard ratio per 1 standard deviation [0.09], 1.26; 95% confidence intervals, 1.08-1.48; *P* = 0.004) and POAG (1.11; 1.01-1.22; *P* = 0.03). The addition of FunSI led to a much clearer improvement in the concordance index for the rhegmatogenous RD model compared with the POAG model.

**Conclusions:**

Fundus Stretch Index is an explainable approach to characterizing myopia at the retinal level. Two individuals may share similar baseline risk factors such as SER, but an overall geometric difference in their fundi, as quantified by FunSI, may reveal a difference in their long-term risk of sight-threatening diseases.

**Financial Disclosures:**

Proprietary or commercial disclosure may be found in the Footnotes and Disclosures at the end of this article.

While increasing myopia is generally accompanied by retinal imaging changes suggestive of a progressively more stretched appearance (e.g., less tortuous and less densely branched vessels),^1^, ^2^, ^3^ some fundi appear far more or far less stretched than would be expected for their degree of myopia. Such discrepancies are partly attributable to the limitation of conventional measures of myopia, which focus on the axial dimension of the eye (visual axis), to describe what is fundamentally a three-dimensional process: ocular growth.^4^ Two eyes may have identical spherical equivalent refraction (SER) yet differ markedly in their posterior segment shape, giving rise to different off-axis retinal morphology.^4^

An interesting question arising from this brief discussion is whether quantitative information from across an individual’s fundus can be incorporated into SER to develop a more individualized, “retinal measure” of myopia.^1^ One approach is to use deep learning (DL) to predict SER directly from a color fundus photograph. The premise is that, assuming a good bias-variance trade-off, a DL model will have a more negative prediction error when applied to a fundus that appears more myopic than expected for the eye’s SER.^5^ This is analogous to how an “older appearing” fundus may indicate accelerated aging (biological age > chronological age).^6^^,^^7^ One such DL-driven retinal measure of myopia is fundus refraction offset (FRO), which has been shown to be predictive of future retinal detachment (RD), independently of baseline SER and relevant covariates.^5^^,^^8^ However, a downside of this approach is its reduced explainability, as the measure is derived directly from raw fundus photographs instead of some readily interpretable imaging features (e.g., vessel tortuosity).

To improve clinical explainability, it may help to consider an alternative approach that has proved very useful in pediatrics growth chart.^9^ At its most basic level, a growth chart displays various centiles of a physical parameter, such as height, as a function of age. A child positioned on the 98th centile, for example, is unusually tall for his/her age (98% of children of the same age are shorter). By substituting height with a fundus imaging feature and age with SER, one could similarly create a fundus centile chart to determine how typical, or atypical, an eye is for its SER. With several such centile charts, each based on a different imaging feature, it may be possible to get an overall picture of how quantitatively different a fundus is relative to other eyes with similar SER. Here, the author introduced an explainable retinal measure of myopia based on this concept, Fundus Stretch Index (FunSI), and investigated its longitudinal association with rhegmatogenous RD and primary open-angle glaucoma (POAG), beyond the influence of baseline covariates, including SER.^10^

## Methods

### Study Participants

This study was reported in accordance with the Strengthening the Reporting of Observational Studies in Epidemiology guidelines.^11^ The study participants were sourced from the UK Biobank Eye and Vision dataset, a large cohort of mid-life (40–69 years) adults recruited from various sites across England, Scotland, and Wales. As the UK Biobank has Research Tissue Bank approval from the Northwest Multi-Center Research Ethics Committee (06/MRE08/65), no separate ethical clearance was required. All participants provided informed consent, and the study adhered to the Declaration of Helsinki.

The reader is referred to previous publications for a detailed description of this cohort.^12^^,^^13^ Briefly, between 2009 and 2010, 68 508 individuals participated in a range of baseline physical and ophthalmic assessments, including 45° macula-centered color fundus photography (Topcon 3D OCT-1000 Mark II) and autorefraction (Tomey RC-5000). In addition to in-person follow-up visits every few years, each individual was linked to the national death registries (2006-2022 for all 3 UK constituent countries) and routinely collected health-related datasets from the UK publicly funded health care system, the National Health Service. These included primary care (1938–2016 for England, 1939–2017 for Scotland, and 1948–2017 for Wales) and hospital admission/procedural records (1981–2022 for Scotland, 1991–2022 for Wales, and 1997–2022 for England).

### Eligibility Criteria

**Figure 1:**
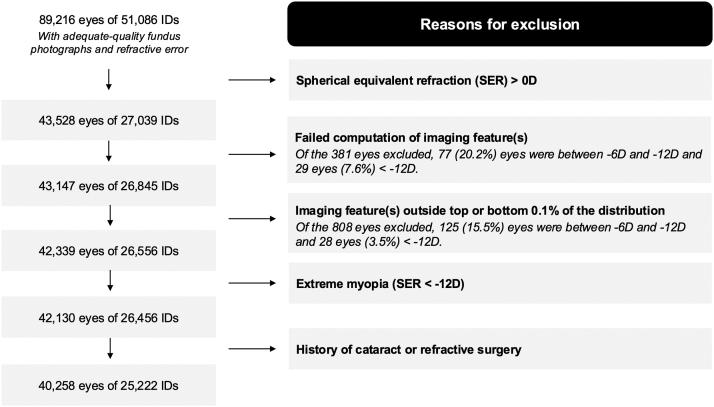
Participant flow diagram. SER = spherical equivalent refraction.

Figure 1 summarizes the flow of participants through each stage of the selection process. Initially, 51 086 individuals with at least 1 eye passing a previously described and validated image quality assessment were included.^1^^,^^14^ Eyes with SER > 0D were subsequently excluded, and those in which one or more imaging features failed computation due to poor image segmentation were further removed. Likewise, eyes lying outside the top and bottom 0.1% of the distribution of any imaging features were excluded, as these outliers were caused by poor image segmentation upon visual inspection.

As very few eyes (n = 209) had extreme myopia above 12 D, centile estimates (described in the next subsection) could not be obtained precisely beyond this level of myopia; these eyes were therefore removed. To rule out the influence of pseudophakia, the author also excluded individuals with a self-reported history of cataract/refractive surgery (UK Biobank data-fields 5324 and 5325) or those with a 3-digit procedural code related to lens extraction based on the Office of Population Censuses and Surveys: Classification of Surgical Operations Version 4, namely C71, C72, and C74, on or prior to the baseline visit. Following this, 40 258 eyes of 25 222 individuals were finally included.

### Centile-Based Fundus Stretch Index

A range of fundus imaging features were computed using automated pipelines described in previous work.^1^^,^^15^ To mitigate the optical influence of ocular magnification and to avoid assuming any specific camera design (e.g., telecentricity) or resolution (mm per pixel),^16^ only dimensionless imaging features were considered when calculating FunSI. These features (n = 10) are summarized in Table 1 and further detailed elsewhere.^1^^,^^15^

**Table 1:** Description of Each Dimensionless (Unitless) Fundus Imaging Feature Used to Derive Fundus Stretch Index

Fundus Feature	Description
Arterial fractal dimension	Complexity of retinal arterioles. Larger values indicate increased complexity (more densely branched vasculature). Generally decreases with myopia.
Venous fractal dimension	Complexity of retinal venules. Larger values indicate increased complexity (more densely branched vasculature). Generally decreases with myopia.
Arteriovenous ratio	Ratio of central retinal arteriolar equivalent to central retinal venular equivalent. Larger values indicate relative arteriolar widening. Generally decreases with myopia.
Arterial tortuosity	Tortuosity of retinal arterioles. Larger values indicate more tortuous arterioles. Generally decreases with myopia.
Venous tortuosity	Tortuosity of retinal venules. Larger values indicate more tortuous venules. Generally decreases with myopia.
Arterial concavity	Parabolic course of the major temporal arterial arcade. Larger values indicate increased concavity (arterial arcade curves more inwardly toward the fovea). Generally increases with myopia.
Venous concavity	Parabolic course of the major temporal venous arcade. Larger values indicate increased concavity (venous arcade curves more inwardly toward the fovea). Generally increases with myopia.
Disc–fovea distance to disc major axis length ratio	Ratio of the Euclidean distance between the optic disc centroid and fovea (disc–fovea distance) to the∗ major axis length of the optic disc. A value of 3, for example, indicates that the disc–fovea distance is 3 times that of the major axis length of the optic disc. Generally increases with myopia.
Disc tilt	Ratio of the∗ major axis length of the optic disc to its∗ minor axis length. Larger values indicate a less circular, more oval optic disc appearance. Generally increases with myopia.
Absolute disc torsion (also known as disc orientation)	Absolute angle between horizontal axis of the image passing through the optic disc centroid and the∗ major axis of the optic disc (ranges from 0° to 90°). A vertically oriented optic disc will have a value of 90°. Generally decreases (less vertically orientated) with myopia.

The reader is kindly referred to Yii et al^1^ for further information about each imaging feature.

∗ Based on the best-fitting ellipse.

**Figure 2:**
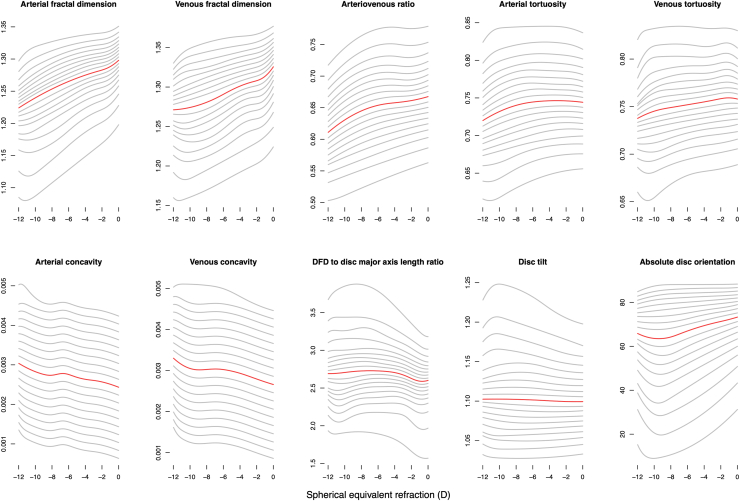
Fundus centile charts, 1 per imaging feature. Each chart consists of 19 centile curves, ranging from the fifth centile (very small value) to the 95th centile (very large value) in 5-centile increments. Centile curve corresponding to the 50th centile (median) is highlighted in red. DFD = disc–fovea distance.

Quantile regression with noncrossing constraints^17^^,^^18^ was used to estimate the 5th to 95th centiles (in 5-centile increments) of each imaging feature, conditioning on SER and using data from 1 randomly selected eye when both eyes of an individual were available. As shown in Figure 2, this resulted in 10 centile charts (1 per imaging feature), each containing 19 centile curves. A key advantage of quantile regression is that it makes no assumptions about the conditional distribution of the dependent variable, in contrast to the less flexible semiparametric Lambda-Mu-Sigma method.

The FunSI for each of the 40 258 eligible eyes was calculated by summing its SER-specific centile position across the 10 centile charts per equation (1), followed by min-max normalization of this sum to a range of 0-1 per equation (2). A FunSI of 0.5 would therefore suggest an “average-looking” fundus given the eye’s SER, while a fundus consistently ranked in the worst centile across all imaging features would have a FunSI of 1. For each imaging feature, the worst centile was either the fifth or the 95th centile, depending on the direction of change of that feature as myopia increased. For example, the worst centile for arterial tortuosity was the fifth centile because tortuosity generally decreased with myopia and vice versa for arterial concavity.^1^

[1] $$Cent_{sum}=Cent_{arterial\;concavity}+Cent_{venous\;concavity}+Cent_{DFD:DML}+Cent_{disc\;tilt}+\left(1–Cent_{arterial\;FD}\right)+\left(1–Cent_{venous\;FD}\right)+\left(1–Cen_{AVR}\right)+\left(1–Cent_{arterial\;tortuosity}\right)+\left(1–Cent_{venous\;tortuosity}\right)+\left(1–Cent_{absolute\;disc\;torsion}\right)$$

[2] $$FunSI=\left(Cent_{sum}\;–\;0.5\right)\;/\;9$$

**Figure 3:**
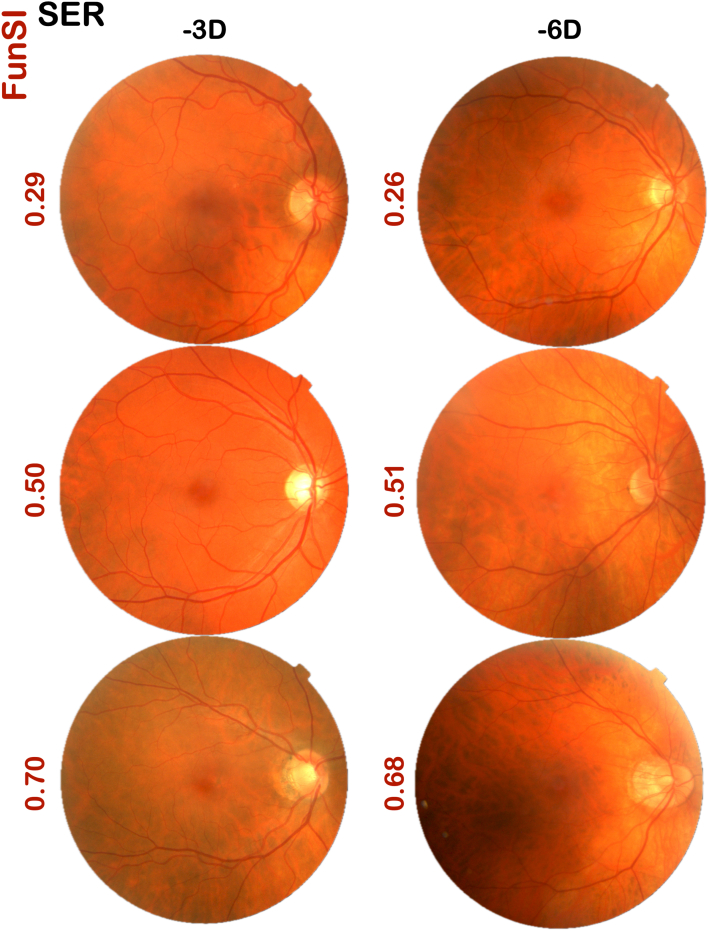
Examples of color fundus photographs with similar SER but different FunSI. Reproduced by kind permission of UK Biobank. SER = spherical equivalent refraction; FunSI = Fundus Stretch Index.

Where *Cent* represents the decimal centile position (e.g., 0.5 for the 50th centile), determined by identifying the centile curve closest to the observed value of a given imaging feature (capped at the 5th and 95th centiles); DFD:DML represents the ratio of disc–fovea distance to disc major axis length; FD represents fractal dimension; and AVR represents arteriovenous ratio. Because the fifth centile (0.05) represented the worst centile for arterial/venous fractal dimension, arteriovenous ratio, arterial/venous tortuosity and absolute disc torsion, subtracting *Cent* corresponding to these imaging features from 1 ensured that a FunSI closer to 1 reflected a more structurally myopic retina. Figure 3 shows some examples of eyes with similar SER but different FunSI.

### Events of Interest

Using a combination of sources, including National Health Service records mentioned earlier and touch-screen questionnaires administered during each assessment visit (self-reported history), the UK Biobank team has made the first-occurrence date of RD/breaks and glaucoma available as data-fields 131178 and 131186, respectively.^19^ However, these were mapped to the first three characters of the International Classification of Diseases version 10 (ICD-10) codes, encompassing various subtypes of RD/breaks (H33) and glaucoma (H40). The source hospital records of individuals with RD/breaks (H33) based on data-field 131178 were accessed to ascertain cases of rhegmatogenous RD (H33.0), given that patients with rhegmatogenous RD would almost certainly require hospital treatment. For POAG, an elimination approach was applied by defining it as the presence of glaucoma (H40) according to data-field 131186 but without (1) any ICD-10 codes related to primary angle-closure glaucoma (H40.2), secondary glaucoma (H40.3 to H40.6), other glaucoma (H40.8), and glaucoma in other diseases (H42.0 and H42.8); as well as (2) any Office of Population Censuses and Surveys: Classification of Surgical Operations Version 4 codes related to angle-closure glaucoma, namely iridosclerotomy (C62.1) and iridotomy (C62.2 and C62.3).

### Populations at Risk and Observation Period

For the analyses described below, the population at risk for rhegmatogenous RD comprised individuals with no prior history of RD/breaks of any subtype (e.g., rhegmatogenous, tractional, and serous), by excluding those with an ICD-10 code H33 (data-field 131178) recorded on or before the baseline visit. Likewise, the population at risk for POAG comprised individuals without glaucoma of any subtype (including angle-closure glaucoma) recorded on or before the baseline visit (ICD-10 code H40; data-field 131186). The observation period (follow-up time) spanned from an individual’s baseline visit (2009–2010) to the right-censoring date, defined as the earliest of last day of 2022, date of death, or date of loss to follow-up due to emigration or withdrawal of consent for future data linkage. For rhegmatogenous RD analyses, the observation period was additionally right-censored at cataract surgery, given the potential confounding effect of cataract surgery during follow-up.^20^ To account for competing risks, the observation period was also right censored at the first occurrence of nonrhegmatogenous RD (for rhegmatogenous RD analyses) or glaucoma unrelated to POAG (for POAG analyses). No individual had zero follow-up time.

### Statistical (Survival) Analysis

All statistical analyses were performed at the individual level because laterality information was not available for the events, averaging continuous data across eyes when both eyes of an individual were eligible (unless otherwise stated).

Kaplan–Meier plots were used to visualize the cumulative incidence ([1-survival probability] Χ 100) of each event over time, stratified by baseline FunSI (categorized into 4 quantiles for visualization purposes). Multivariable Cox regression models were then fit to test the association between baseline FunSI (continuous) and time to each event, defined as the time elapsed from the baseline visit to the first-occurrence date or the right-censoring date (whichever came first). Note that FunSI was standardized to zero mean and unit variance in all models to place the estimated hazard ratio (HR) on a less steep and more interpretable scale, such that an HR of x represented an x-fold change in hazard rate for every 1 standard deviation (SD) change in FunSI, rather than per 1-unit change in the index (which would unrealistically reflect a change across the full 0-1 theoretical range of FunSI). The models initially assumed a linear relationship and were then extended to include nonlinear FunSI terms: a second-degree polynomial (FunSI + FunSI^2^), followed by a third-degree polynomial (FunSI + FunSI^2^ + FunSI^3^).

### Covariate Adjustment

Baseline covariates selected a priori and adjusted for in each model included basic/common demographic and clinical information: age (continuous), sex (binary), SER (continuous: spherical power + cylindrical power/2), Townsend deprivation index (continuous: positive values indicated higher deprivation), ethnicity (binary: White vs. non-White), diabetes (Boolean), and hypertension (Boolean). History of ocular trauma (Boolean) was additionally included in the rhegmatogenous RD model, while intraocular pressure (IOP) and corneal hysteresis (CH),^21^ both of which were continuous variables measured with the Reichert Ocular Response Analyser, were additionally included in the POAG model. Unreasonable IOP (0 or >45 mmHg)^22^ and CH (0 or >15 mmHg)^23^, ^24^, ^25^ values were removed (treated as missing), as these were most likely caused by measurement error. The reader is referred to Yii et al^8^ for further information about each covariate.

### Leave-One-Feature-Out, Sensitivity, and Subgroup Analyses

To assess the relative influence of each imaging feature on the Cox regression results, FunSI was recomputed using a leave-one-feature-out approach (1 feature omitted at a time). Additionally, sensitivity analyses were conducted by (1) excluding self-reported cases and cases not identified through hospital records (this analysis was not applicable to rhegmatogenous RD because, by definition, all cases had a hospital record); (2) excluding individuals with cylindrical power >2D to rule out the potential influence of refractive myopia (e.g., due to keratoconus); (3) excluding cases occurring within 1 year of baseline to rule out any inadvertent inclusion of prevalent cases resulting from a potential time lag between disease onset and the availability of records; (4) selecting FunSI and covariate data from the more myopic eye, when both eyes were eligible; and (5) lowering the imaging feature outlier threshold (as described under the eligibility criteria) from 0.1% to 0.01% (thus excluding fewer eyes). Finally, in a subgroup of individuals not previously used to train the FRO DL model,^5^ baseline FRO was included as an additional continuous covariate (subgroup analysis).

The variance inflation factor was <1.2 in all fitted models, indicating no evidence of multicollinearity. A complete-case approach was used to handle missing data, when the data could be assumed to be missing completely at random, based on Little’s test.^26^^,^^27^ Otherwise, multiple (n = 10) imputation using the predictive mean matching method^28^ was performed. The concordance index (C-index) was used to summarize the discriminative ability of the fitted models, with higher values indicating better discrimination.^29^ All analyses were performed in R V.4.5.1 (R Core Team), using the *quantregGrowth* and *survival* packages for centile curve estimation and survival analyses, respectively. The source code is openly available at https://github.com/fyii200/FunSI. Hypothesis testing was 2-tailed, with *P* < 0.05 considered sufficient evidence to reject the null hypotheses.

## Results

### Baseline Characteristics and Cumulative Incidence

Table 2 summarizes the baseline characteristics of the populations at risk for rhegmatogenous RD and POAG. For rhegmatogenous RD, 25 030 (99.9%) of 25 067 at-risk individuals had complete data; 37 individuals with missing Townsend deprivation index were excluded from all analyses, as the missing completely at random assumption was met (χ^2^ = 6.4; *P* = 0.79). For POAG, 23 600 (95.0%) of 24 835 at-risk individuals had complete data; missing values for CH (n = 1191), IOP (n = 750), and Townsend deprivation index (n = 37) were imputed, as the missing completely at random assumption was violated (χ^2^ = 584.4; *P* < 0.001).

**Table 2:** Baseline Characteristics of Participants Included in the Survival Analyses

Characteristics	Rhegmatogenous RD (n = 25 030)	Primary Open-Angle Glaucoma (n = 24 835)
Age (year)	54.3 (8.1)	54.3 (8.1)
Female	13 513 (54.0)	13 430 (54.1)
White	22 742 (90.9)	22 572 (90.9)
SER (diopter)	–2.12 (2.23)	–2.12 (2.23)
FunSI	0.50 (0.09)	0.50 (0.09)
Townsend deprivation index	–1.03 (2.95)	–1.04 (2.95)
Diabetes	1107 (4.4)	1080 (4.3)
Hypertension	5502 (22.0)	5440 (21.9)
Ocular trauma	124 (0.5)	/
Intraocular pressure (mmHg)	/	15.9 (3.5)
Corneal hysteresis (mmHg)	/	10.5 (1.7)

Continuous variables are presented as mean (standard deviation) and categorical variables as n (%).

RD = retinal detachment; SER = spherical equivalent refraction; FunSI = Fundus Stretch Index.

The mean (SD) observation period was 12.0 (2.1) for rhegmatogenous RD (301 348 person-years) and 12.3 (1.6) years for POAG (306 179 person-years). A total of 148 individuals had incident rhegmatogenous RD during the observation period, yielding an event rate of 4.9 cases per 10 000 person-years. The number of incident POAG cases was 460, corresponding to an event rate of 15.0 cases per 10 000 person-years. Of these POAG cases, 23 were self-reported, and 437 were identified through other sources, including 319 through hospital records.

**Figure 4:**
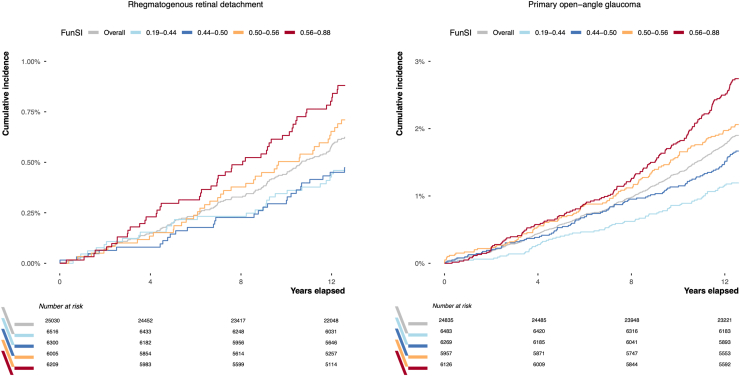
Cumulative incidence (calculated as [1 - Kaplan–Meier survival probability] Χ 100) of rhegmatogenous retinal detachment and primary open-angle glaucoma over time, stratified by baseline FunSI (categorized into 4 quantiles for visualization purposes). FunSI = Fundus Stretch Index.

The cumulative incidence of each event over time, stratified by baseline FunSI, is shown in Figure 4. Compared with individuals in the lowest FunSI quantile, those in the highest FunSI quantile had twice as high cumulative incidence of rhegmatogenous RD over 12 years (0.8% [95% confidence interval {CI}: 0.6%–1.1%] vs. 0.4% [0.3%–0.6%]). Similarly, the 12-year cumulative incidence of POAG was more than twice as high in the highest FunSI quantile (2.5% [2.1%–2.9%]) as that observed in the lowest FunSI quantile (1.2% [0.9%–1.4%]).

### Multivariable Cox Regression

As shown in Table 3, higher baseline FunSI was associated with an increased risk of developing rhegmatogenous RD (adjusted HR per 1 SD [0.09], 1.26; 95% CI, 1.08–1.48; *P* = 0.004) and POAG (1.11; 1.01–1.22; *P* = 0.03), even after adjusting for baseline covariates including SER (univariable associations are presented in Table S1, available at www.ophthalmologyscience.org). The C-index for the rhegmatogenous RD model without FunSI was 0.689, increasing by 0.017 to 0.706 when FunSI was added. In contrast, the addition of FunSI to the POAG model only led to a very modest increase in the C-index (+0.003, from 0.804 to 0.807).

**Table 3:** Adjusted Association of Baseline FunSI and Covariates with Each Event (Multivariable Cox Regression)

Baseline Variables	Rhegmatogenous RD (n = 25 030)		Primary Open-Angle Glaucoma (n = 24 835)	
	Adjusted HR (95% CI)	*P*	Adjusted HR (95% CI)	*P*
SER, per 1 diopter	0.81 (0.77 to 0.85)	<0.001	0.93 (0.89 to 0.96)	<0.001
FunSI, per 1 SD (0.09)	1.26 (1.08 to 1.48)	0.004	1.11 (1.01 to 1.22)	0.03
Age, per 1 year	1.00 (0.98 to 1.02)	0.83	1.07 (1.05 to 1.08)	<0.001
Male sex	1.89 (1.35 to 2.63)	<0.001	1.14 (0.95 to 1.38)	0.16
Townsend deprivation index, per 1 unit	0.97 (0.92 to 1.03)	0.39	0.99 (0.96 to 1.02)	0.55
White ethnicity	1.22 (0.63 to 2.35)	0.56	0.66 (0.48 to 0.90)	0.01
Diabetes	1.05 (0.48 to 2.30)	0.89	1.58 (1.12 to 2.22)	0.009
Hypertension	1.06 (0.71 to 1.57)	0.78	1.12 (0.91 to 1.38)	0.27
Ocular trauma	2.35 (0.58 to 9.50)	0.23	/	/
IOP, per 1 mmHg	/	/	1.21 (1.19 to 1.23)	<0.001
CH, per 1 mmHg	/	/	0.91 (0.86 to 0.96)	0.001

RD = retinal detachment; HR = hazard ratio; CI = confidence interval; SER = spherical equivalent refraction; FunSI = Fundus Stretch Index; SD = standard deviation; IOP = intraocular pressure; CH = corneal hysteresis.

### Addition of Nonlinear Terms

None of the nonlinear FunSI terms were associated with the risk of rhegmatogenous RD, whether modeled as a second-degree polynomial (*P* = 0.39 for FunSI^2^) or a third-degree polynomial (*P* = 0.55 for FunSI^2^ and *P* = 0.19 for FunSI^3^). There was some weak indication of a quadratic relationship between FunSI and POAG risk: in the second-degree polynomial model, the FunSI^2^ term was associated with disease risk (HR, 0.90; 95% CI, 0.82–1.00; *P* = 0.046), but its inclusion did not improve the C-index (0.806, similar to the original linear model).

### Leave-One-Feature-Out, Sensitivity, and Subgroup Analyses

Findings from the leave-one-feature-out analysis are presented in Table S2 (available at www.ophthalmologyscience.org). Baseline FunSI remained associated (*P* < 0.05) with rhegmatogenous RD risk regardless of which imaging feature was removed, with estimated HRs ranging from 1.19 to 1.30. For POAG, however, baseline FunSI was no longer associated with disease risk after removing *DFD:DML*, arterial/venous fractal dimension, or arteriovenous ratio, suggesting that these features had a disproportionate influence on FunSI’s association with POAG risk.

The overall conclusions—that higher baseline FunSI was associated with an increased risk of rhegmatogenous RD and POAG—remained unchanged after repeating the multivariable Cox regression across all 5 sensitivity analyses: (1) exclusion of self-reported and nonhospital cases (Table S3, available at www.ophthalmologyscience.org); (2) exclusion of individuals with cylindrical power >2D (Table S4, available at www.ophthalmologyscience.org); (3) exclusion of cases occurring within 1 year of baseline (Table S5, available at www.ophthalmologyscience.org); (4) analysis of data from the more myopic eye (Table S6, available at www.ophthalmologyscience.org); and (5) lowering the imaging feature outlier threshold to 0.01% (Table S7, available at www.ophthalmologyscience.org).

In the subgroup analysis, 104 of 15 954 eligible individuals had incident rhegmatogenous RD, and 335 of 15 754 had incident POAG. As shown in Table S8 (available at www.ophthalmologyscience.org), baseline FRO (adjusted HR per 1 SD [0.87 D], 0.77; 95% CI, 0.65–0.90; *P* = 0.002) and FunSI (1.29; 1.07 to 1.57; *P* = 0.009) were both independently associated with the risk of developing rhegmatogenous RD. The C-index was 0.679 for the rhegmatogenous RD model without FRO and FunSI, increasing by 0.021 to 0.700 after adding FRO, and further increased by 0.018 to 0.718 with the inclusion of FunSI—an overall improvement of almost 0.04. For POAG, there was insufficient evidence to suggest that FRO (0.91; 0.82–1.00; *P* = 0.053) and FunSI (1.09; 0.98–1.22; *P* = 0.10) were independently associated with disease risk, although some trends were observed. Accordingly, the changes in the C-index attributable to FRO (+0.001) and FunSI (+0.001) were practically absent, with a negligible overall improvement of 0.002 from 0.796 to 0.798.

## Discussion

The present study introduced FunSI, a retinal measure of myopia that summarizes an eye’s SER-specific centile position across a range of explainable fundus imaging features (Table 1), where a higher value suggests a more stretched fundus relative to the eye’s SER. As demonstrated in the primary analysis, individuals with similar SER and other baseline risk factors may nonetheless exhibit geometric differences in their fundi, as summarized by FunSI, which may translate into differences in the risk of developing rhegmatogenous RD (or, to a lesser extent, POAG) over the subsequent 12 years.

In the subgroup analysis, both baseline FunSI and FRO^5^^,^^8^ were found to be associated with the risk of developing rhegmatogenous RD, independently of one another. Indeed, a post hoc linear regression of FRO on FunSI showed that, while a higher FunSI was associated (*P* = 0.01) with a more negative FRO (more myopic-looking fundus as inferred by DL), the strength of association was weak, where a 1 SD increase in FunSI was only associated with –0.02 D (95% CI, –0.03 D to –0.003 D) change in FRO. Both measures are thus complementary, as they appear to capture different retinal information pertinent to myopia. However, when considering POAG as the event in the subgroup analysis, both FRO and FunSI were not associated with the disease, although some trends were observed. Compared with rhegmatogenous RD (Table 3), the association between FunSI and POAG in the primary analysis also appeared weaker (HR: 1.26 vs. 1.11) and more uncertain, with the lower bound of 95% CI closer to the null. Additionally, the improvement in the C-index attributable to FunSI was substantially smaller for the POAG model than for the rhegmatogenous RD model, in both the primary and subgroup analyses. Taken together, the evidence supporting FunSI (in its current form) as an independent risk marker for POAG is comparatively weaker.

A precursor to rhegmatogenous RD is the presence of vitreoretinal tractional forces acting on the neurosensory retina,^30^^,^^31^ which, as previously discussed,^8^ may affect fundus appearance. Even when such tractional forces are not yet clinically evident, signs of strain imparted by an axially elongated, aspheric (more prolate or less oblate) posterior segment characteristic of a myopic eye may already be detectable, however, subtly, at the pixel level across the fundus,^4^ changes that could predispose the eye to rhegmatogenous RD.^32^ Signs of myopic strain involving the optic nerve head (e.g., tilted and more obliquely orientated disc) and retinal vasculature (e.g., reduced fractal dimension) are also implicated in the increased susceptibility of myopic eyes to POAG, either mediated by a mechanical pathway or a perfusion-related pathway or both.^33^, ^34^, ^35^, ^36^

Fundus Stretch Index and, for that matter, FRO, differ from SER in being adjunctive measures of myopia that are more directly concerned with the structural characteristics of the retina. This emphasis may seem pedantic, but it closely aligns with recent high-profile consensus statements recommending that myopia be classified as a disease, first by the US National Academies of Science^37^ and subsequently by the International Myopia Summit Workgroup.^38^ The latter workgroup identified, as a “key strategy” for reducing the global burden of myopia, the need to define myopia not solely as a refractive error but a disease with phenotypic features that influence long-term eye health outcomes.^38^ This paradigm shift is an important step toward refining risk stratification in clinical practice,^38^ as some patients with low myopia are at high risk of developing complications, far in excess of what is typical for their SER, whereas some highly myopic patients do not develop such complications. In practice, both FunSI and FRO provide the first proof of concept to advance this paradigm shift.

The male predilection to rhegmatogenous RD in this study (Table 3) is consistent with previous epidemiological findings.^39^ Likewise, diabetes^40^ and higher IOP^41^ are two important risk factors for POAG, and mounting longitudinal evidence supports lower CH (reflecting reduced ability of ocular tissue to dissipate energy) as an independent risk factor for POAG onset,^42^ including a recent UK Biobank study.^43^

Despite the use of a large-scale, population-based dataset, the fundus centile charts were derived from color fundus photographs acquired with a Topcon camera. Although some degree of generalizability to other cameras may be expected, given that only dimensionless imaging features were considered, this requires external validation in future work. Likewise, further work is warranted to establish if the current version of FunSI is transferable across populations, given the predominance of a single ethnic group (White European) in the UK Biobank. As a proof of concept, this study lays the groundwork for other researchers to develop their own FunSI, with the potential for combining derived data in the future to create cross-population fundus centile charts. Another limitation is that it remains unclear whether FunSI is an independent measure of myopia or a complex surrogate for axial length due to the absence of axial length measurements in the UK Biobank. In addition to the need for external validation and adjustment for axial length, future work could incorporate additional relevant imaging features, such as cup-to-disc ratio and degree of tessellation, to potentially improve the potential of FunSI for risk stratification. It would also be valuable for future longitudinal studies to investigate the association between FunSI and the risk of pathologic myopia or childhood myopia. Another interesting line of inquiry is to disentangle the potential relationships among FunSI, height, three-dimensional eye shape, and eye volume.

In conclusion, FunSI is a promising, explainable retinal measure of myopia that may complement FRO in stratifying the risk of future rhegmatogenous RD—and, to a lesser extent, POAG—even among individuals with similar SER, age, sex, and other baseline risk factors.
