# Supplementary material for: Fundus Stretch Index: A Centile-Based Retinal Measure of Myopia
Source: Ophthalmol Sci. 2026 Mar 18;6(5):101162. doi: 10.1016/j.xops.2026.101162 (PMC13099463; doi:10.1016/j.xops.2026.101162)
Supplement: Supplementary Tables [file mmc1.pdf]

Supplemental Online Content

Supplementary	Brief description	Page
**Table S1**	Univariable association of Fundus Stretch Index and potential covariates with each event.	2
**Table S2**	Leave-one-feature-out analysis	3
**Table S3**	Sensitivity analysis 1: self-reported and non-hospital cases were excluded.	4
**Table S4**	Sensitivity analysis 2: individuals with cylindrical power >2D were excluded.	5
**Table S5**	Sensitivity analysis 3: cases occurring within one year of the baseline visit were excluded.	6
**Table S6**	Sensitivity analysis 4: data from the more myopic eye were analysed when both eyes were eligible.	7
**Table S7**	Sensitivity analysis 5: threshold for excluding outlying imaging feature values was lowered from 0.1% to 0.01%.	8
**Table S8**	Subgroup analysis: Fundus Refraction Offset was included as another covariate.	9

Table S1. Univariable (unadjusted) association of baseline FunSI and covariates with each event.

Baseline variables	**Rhegmatogenous RD** (n=25,030)		**Primary open-angle glaucoma** (n=24,835)	
	Unadjusted HR (95% CI)	*P*	Unadjusted HR (95% CI)	*P*
SER, *per 1 dioptre*	0.81 (0.77 to 0.86)	<.001	0.91 (0.88 to 0.94)	<.001
FunSI, *per 1 SD (0.09)*	1.28 (1.10 to 1.50)	.002	1.34 (1.22 to 1.46)	<.001
Age, *per 1 year*	1.01 (0.99 to 1.03)	.20	1.09 (1.08 to 1.11)	<.001
Male sex	1.83 (1.32 to 2.55)	<.001	1.39 (1.16 to 1.68)	<.001
White ethnicity	1.39 (0.73 to 2.65)	.31	0.88 (0.65 to 1.20)	.43
Townsend deprivation index, *per 1 unit*	0.97 (0.91 to 1.02)	.26	0.97 (0.94 to 1.01)	.12
Diabetes	1.18 (0.55 to 2.52)	.67	2.20 (1.59 to 3.04)	<.001
Hypertension	1.20 (0.83 to 1.75)	.34	1.71 (1.41 to 2.08)	<.001
Ocular trauma	2.78 (0.69 to 11.21)	.15	/	/
IOP, *per 1 mmHg*	/	/	1.24 (1.22 to 1.26)	<.001
CH, *per 1 mmHg*	/	/	0.84 (0.79 to 0.89)	<.001

RD, *retinal detachment*; HR, *hazard ratio*; CI, *confidence intervals*; SER, *spherical equivalent refraction*; FunSI, *Fundus Stretch Index*; SD, *standard deviation*; IOP, *intraocular pressure*; CH, *corneal hysteresis*

Table S2. Adjusted association between leave-one-feature-out baseline FunSI and the risk of each event (each row corresponds to a fitted multivariable Cox regression model).

Baseline FunSI	**Rhegmatogenous RD** (n=25,030)		**Primary open-angle glaucoma** (n=24,835)	
	[a]Adjusted HR (95% CI)	*P*	[b]Adjusted HR (95% CI)	*P*
Without arterial concavity *per 1 SD (0.10)*	1.29 (1.10 to 1.51)	.002	1.12 (1.02 to 1.22)	.02
Without arterial FD *per 1 SD (0.09)*	1.19 (1.02 to 1.40)	.03	1.08 (0.99 to 1.18)	.10
Without arterial tortuosity *per 1 SD (0.10)*	1.30 (1.11 to 1.53)	.001	1.12 (1.02 to 1.23)	.02
Without arteriovenous ratio *per 1 SD (0.10)*	1.20 (1.02 to 1.40)	.03	1.10 (1.00 to 1.20)	.050
Without DFD:DML *per 1 SD (0.09)*	1.27 (1.08 to 1.49)	.004	1.03 (0.94 to 1.14)	.47
Without disc tilt *per 1 SD (0.10)*	1.29 (1.09 to 1.51)	.003	1.13 (1.03 to 1.25)	.008
Without absolute disc torsion *per 1 SD (0.10)*	1.22 (1.04 to 1.43)	.02	1.11 (1.01 to 1.22)	.02
Without venous concavity *per 1 SD (0.10)*	1.25 (1.07 to 1.47)	.006	1.14 (1.03 to 1.25)	.007
Without venous FD *per 1 SD (0.09)*	1.23 (1.05 to 1.44)	.01	1.07 (0.98 to 1.17)	.16
Without venous tortuosity *per 1 SD (0.10)*	1.28 (1.09 to 1.50)	.003	1.16 (1.05 to 1.27)	.002

RD, *retinal detachment*; HR, *hazard ratio*; CI, *confidence intervals*; FunSI, *Fundus Stretch Index*; SD, *standard deviation*; FD, *fractal dimension*; DFD:DML, ratio of disc-fovea distance to disc major axis length

[a]Adjusted for baseline age, sex, spherical equivalent refraction, Townsend deprivation index, ethnicity, diabetes, hypertension and ocular trauma

[b]Adjusted for baseline age, sex, spherical equivalent refraction, Townsend deprivation index, ethnicity, diabetes, hypertension, intraocular pressure and corneal hysteresis

Table S3. Adjusted association between baseline FunSI and the risk of primary open-angle glaucoma after excluding 23 self-reported cases and 118 non-hospital cases, leaving 319 cases (sensitivity analysis 1).

Baseline variables included in multivariable Cox regression	**Primary open-angle glaucoma** (n=24,694)	
	Adjusted HR (95% CI)	*P*
SER, *per 1 dioptre*	0.93 (0.89 to 0.97)	.001
FunSI, *per 1 SD (0.09)*	1.16 (1.04 to 1.30)	.008
Age, *per 1 year*	1.10 (1.08 to 1.12)	<.001
Male sex	1.04 (0.83 to 1.31)	.70
Townsend deprivation index, *per 1 unit*	1.04 (1.00 to 1.08)	.08
White ethnicity	0.49 (0.35 to 0.70)	<.001
Diabetes	1.47 (0.98 to 2.21)	.07
Hypertension	1.01 (0.79 to 1.30)	.94
IOP, *per 1 mmHg*	1.19 (1.17 to 1.22)	<.001
CH, *per 1 mmHg*	0.89 (0.83 to 0.95)	<.001

HR, *hazard ratio*; CI, *confidence intervals*; SER, *spherical equivalent refraction*; FunSI, *fundus stretch index*; SD, *standard deviation*; *intraocular pressure*; CH, *corneal hysteresis*

Table S4. Adjusted association between baseline FunSI and the risk of each event after excluding individuals with cylindrical power >2D (sensitivity analysis 2).

Baseline variables included in multivariable Cox regression	**Rhegmatogenous RD** (n=23,042)		**Primary open-angle glaucoma** (n=22,882)	
	Adjusted HR (95% CI)	*P*	Adjusted HR (95% CI)	*P*
SER, *per 1 dioptre*	0.80 (0.76 to 0.85)	<.001	0.94 (0.90 to 0.97)	.001
FunSI, *per 1 SD (0.09)*	1.21 (1.02 to 1.45)	.03	1.11 (1.01 to 1.23)	.03
Age, *per 1 year*	1.01 (0.98 to 1.03)	.57	1.07 (1.05 to 1.08)	<.001
Male sex	2.17 (1.50 to 3.15)	<.001	1.17 (0.96 to 1.43)	.12
Townsend deprivation index, *per 1 unit*	0.95 (0.89 to 1.01)	.11	0.99 (0.95 to 1.02)	.45
White ethnicity	0.94 (0.48 to 1.83)	.86	0.62 (0.45 to 0.86)	.005
Diabetes	1.24 (0.57 to 2.73)	.59	1.44 (0.99 to 2.10)	.053
Hypertension	1.06 (0.69 to 1.63)	.79	1.15 (0.92 to 1.43)	.22
Ocular trauma	1.30 (0.18 to 9.34)	.79	/	/
IOP, *per 1 mmHg*	/	/	1.21 (1.19 to 1.23)	<.001
CH, *per 1 mmHg*	/	/	0.92 (0.87 to 0.97)	.002

RD, *retinal detachment*; HR, *hazard ratio*; CI, *confidence intervals*; SER, *spherical equivalent refraction*; FunSI, *fundus stretch index*; SD, *standard deviation*; *intraocular pressure*; CH, *corneal hysteresis*

Table S5. Adjusted association between baseline FunSI and the risk of each event after excluding 8 rhegmatogenous RD cases and 3 primary open-angle glaucoma cases that occurred within 1 year of the baseline visit (sensitivity analysis 3).

Baseline variables included in multivariable Cox regression	**Rhegmatogenous RD** (n=25,022)		**Primary open-angle glaucoma** (n=24,832)	
	Adjusted HR (95% CI)	*P*	Adjusted HR (95% CI)	*P*
SER, *per 1 dioptre*	0.81 (0.76 to 0.85)	<.001	0.93 (0.90 to 0.96)	<.001
FunSI, *per 1 SD (0.09)*	1.31 (1.11 to 1.54)	.001	1.11 (1.01 to 1.22)	.03
Age, *per 1 year*	0.99 (0.97 to 1.02)	.53	1.07 (1.05 to 1.08)	<.001
Male sex	1.84 (1.31 to 2.59)	<.001	1.15 (0.95 to 1.39)	.14
Townsend deprivation index, *per 1 unit*	0.98 (0.92 to 1.04)	.44	0.99 (0.96 to 1.02)	.49
White ethnicity	1.16 (0.60 to 2.24)	.66	0.65 (0.47 to 0.89)	.008
Diabetes	0.96 (0.41 to 2.21)	.92	1.58 (1.12 to 2.22)	.009
Hypertension	1.09 (0.73 to 1.64)	.67	1.12 (0.91 to 1.38)	.29
Ocular trauma	1.23 (0.17 to 8.84)	.84	/	/
IOP, *per 1 mmHg*	/	/	1.21 (1.19 to 1.23)	<.001
CH, *per 1 mmHg*	/	/	0.90 (0.86 to 0.95)	<.001

RD, *retinal detachment*; HR, *hazard ratio*; CI, *confidence intervals*; SER, *spherical equivalent refraction*; FunSI, *fundus stretch index*; SD, *standard deviation*; *intraocular pressure*; CH, *corneal hysteresis*

Table S6. Adjusted association between baseline FunSI and the risk of each event, using FunSI and covariate data from the more myopic eye when both eyes of an individual were eligible (sensitivity analysis 4).

Baseline variables included in multivariable Cox regression	**Rhegmatogenous RD** (n=25,030)		**Primary open-angle glaucoma** (n=24,835)	
	Adjusted HR (95% CI)	*P*	Adjusted HR (95% CI)	*P*
SER, *per 1 dioptre*	0.81 (0.77 to 0.86)	<.001	0.93 (0.90 to 0.97)	<.001
FunSI, *per 1 SD (0.10)*	1.21 (1.03 to 1.42)	.02	1.12 (1.02 to 1.23)	.01
Age, *per 1 year*	1.00 (0.98 to 1.02)	.96	1.07 (1.06 to 1.09)	<.001
Male sex	1.89 (1.36 to 2.64)	<.001	1.18 (0.98 to 1.43)	.08
Townsend deprivation index, *per 1 unit*	0.97 (0.92 to 1.03)	.35	0.99 (0.96 to 1.02)	.53
White ethnicity	1.21 (0.63 to 2.34)	.56	0.62 (0.45 to 0.85)	.003
Diabetes	1.07 (0.49 to 2.33)	.87	1.56 (1.11 to 2.19)	.01
Hypertension	1.07 (0.72 to 1.58)	.75	1.12 (0.91 to 1.38)	.29
Ocular trauma	2.33 (0.57 to 9.42)	.24	/	/
IOP, *per 1 mmHg*	/	/	1.19 (1.17 to 1.21)	<.001
CH, *per 1 mmHg*	/	/	0.92 (0.88 to 0.97)	.002

RD, *retinal detachment*; HR, *hazard ratio*; CI, *confidence intervals*; SER, *spherical equivalent refraction*; FunSI, *fundus stretch index*; SD, *standard deviation*; *intraocular pressure*; CH, *corneal hysteresis*

Table S7. Adjusted association between baseline FunSI and the risk of each event after lowering the threshold for excluding outlying imaging feature values from 0.1% to 0.01% (sensitivity analysis 5).

Baseline variables included in multivariable Cox regression	**Rhegmatogenous RD** (n=25,252)		**Primary open-angle glaucoma** (n=25,052)	
	Adjusted HR (95% CI)	*P*	Adjusted HR (95% CI)	*P*
SER, *per 1 dioptre*	0.81 (0.77 to 0.86)	<.001	0.92 (0.89 to 0.96)	<.001
FunSI, *per 1 SD (0.09)*	1.27 (1.08 to 1.49)	.003	1.10 (1.01 to 1.21)	.04
Age, *per 1 year*	1.00 (0.98 to 1.02)	.85	1.07 (1.05 to 1.08)	<.001
Male sex	1.85 (1.33 to 2.58)	<.001	1.14 (0.95 to 1.38)	.17
Townsend deprivation index, *per 1 unit*	0.97 (0.92 to 1.03)	.35	0.99 (0.96 to 1.02)	.46
White ethnicity	1.22 (0.63 to 2.35)	.55	0.65 (0.48 to 0.90)	.009
Diabetes	1.07 (0.49 to 2.32)	.87	1.57 (1.12 to 2.21)	.009
Hypertension	1.04 (0.70 to 1.55)	.83	1.15 (0.94 to 1.41)	.17
Ocular trauma	2.31 (0.57 to 9.35)	.24	/	/
IOP, *per 1 mmHg*	/	/	1.21 (1.20 to 1.24)	<.001
CH, *per 1 mmHg*	/	/	0.90 (0.86 to 0.95)	<.001

RD, *retinal detachment*; HR, *hazard ratio*; CI, *confidence intervals*; SER, *spherical equivalent refraction*; FunSI, *fundus stretch index*; SD, *standard deviation*; *intraocular pressure*; CH, *corneal hysteresis*

Table S8. Adjusted association of baseline FunSI and FRO with each event (subgroup analysis).

Baseline variables included in multivariable Cox regression		Rhegmatogenous RD (n=15,954)		Primary open-angle glaucoma (n=15,754)	
		Adjusted HR (95% CI)	*P*	Adjusted HR (95% CI)	*P*
SER	*per 1 dioptre*	0.80 (0.75 to 0.86)	<.001	0.93 (0.89 to 0.97)	.001
FunSI	*per 1 SD (0.09)*	1.29 (1.07 to 1.57)	.009	1.09 (0.98 to 1.22)	.10
FRO	*per 1 dioptre*<br>*per 1 SD (0.87 dioptre)*	0.73 (0.61 to 0.89)<br>0.77 (0.65 to 0.90)	.002	0.89 (0.80 to 1.00)<br>0.91 (0.82 to 1.00)	.053
Age	*per 1 year*	0.99 (0.97 to 1.02)	.50	1.07 (1.05 to 1.09)	<.001
Male sex		1.79 (1.21 to 2.65)	.004	1.11 (0.89 to 1.39)	.36
Townsend deprivation index, *per 1 unit*		1.00 (0.93 to 1.07)	.98	1.00 (0.97 to 1.04)	.82
White ethnicity		1.26 (0.58 to 2.77)	.56	0.63 (0.43 to 0.91)	.02
Diabetes		1.31 (0.56 to 3.08)	.53	1.53 (1.04 to 2.25)	.03
Hypertension		1.07 (0.67 to 1.70)	.79	1.20 (0.95 to 1.53)	.13
Ocular trauma		1.44 (0.20 to 10.33)	.72	/	/
IOP	*per 1 mmHg*	/	/	1.21 (1.18 to 1.23)	<.001
CH	*per 1 mmHg*	/	/	0.92 (0.87 to 0.98)	.01

RD, *retinal detachment*; HR, *hazard ratio*; CI, *confidence intervals*; SER, *spherical equivalent refraction*; FunSI, *Fundus Stretch Index*; SD, *standard deviation*; FRO, *Fundus Refraction Offset*; IOP, *intraocular pressure*; CH, *corneal hysteresis*
